# Hypocretin-1/orexin-A fragment_1-16_ as a potential surrogate marker for diagnosing narcolepsy type 1

**DOI:** 10.1093/sleep/zsag115

**Published:** 2026-05-15

**Authors:** Elena S Wenz, Jean-Christophe Prost, Sophie Braga-Lagache, Gabriela M Mäder, Jan D Warncke, Martin Luginbühl, Patrick Y Wuethrich, Friedrich Lersch, Christoph Schankin, Irina Filchenko, Livia Fregolente, Kseniia Zub, the iSPHYNCS consortium, Seiji Nishino, Carlo R Largiadèr, Manfred Heller, Mehdi Tafti, Markus H Schmidt, Claudio L A Bassetti

**Affiliations:** Department of Neurology, Inselspital, Bern University Hospital, University of Bern, Bern, Switzerland; Graduate School of Health Sciences, University of Bern, Bern, Switzerland; Department of Clinical Chemistry, Inselspital, Bern University Hospital, University of Bern, Bern, Switzerland; Department for Biomedical Research, Core Facility Proteomics & Mass Spectrometry, University of Bern, Bern, Switzerland; Department of Clinical Chemistry, Inselspital, Bern University Hospital, University of Bern, Bern, Switzerland; Department of Neurology, Inselspital, Bern University Hospital, University of Bern, Bern, Switzerland; Department of Anaesthesiology and Pain Medicine, Inselspital, Bern University Hospital, University of Bern, Bern, Switzerland; Department of Anaesthesiology and Pain Medicine, Inselspital, Bern University Hospital, University of Bern, Bern, Switzerland; Department of Anaesthesiology and Pain Medicine, Inselspital, Bern University Hospital, University of Bern, Bern, Switzerland; Department of Neurology, Inselspital, Bern University Hospital, University of Bern, Bern, Switzerland; Department of Neurology, Inselspital, Bern University Hospital, University of Bern, Bern, Switzerland; Department of Neurology, Inselspital, Bern University Hospital, University of Bern, Bern, Switzerland; Graduate School of Health Sciences, University of Bern, Bern, Switzerland; Department of Neurology, Inselspital, Bern University Hospital, University of Bern, Bern, Switzerland; Sleep and Circadian Neurobiology Laboratory, School of Medicine, Stanford University, Stanford, CA, United States; Department of Clinical Chemistry, Inselspital, Bern University Hospital, University of Bern, Bern, Switzerland; Department for Biomedical Research, Core Facility Proteomics & Mass Spectrometry, University of Bern, Bern, Switzerland; Department of Biomedical Sciences, University of Lausanne, Lausanne, Switzerland; Department of Neurology, Inselspital, Bern University Hospital, University of Bern, Bern, Switzerland; Department of Neurology, Inselspital, Bern University Hospital, University of Bern, Bern, Switzerland; Medical Faculty, University of Bern, Bern, Switzerland

**Keywords:** narcolepsy type 1, hypocretin, orexin, quantification, cerebrospinal fluid, mass spectrometry, radioimmunoassay, surrogate marker, fragment

## Abstract

**Study Objectives:**

Loss of cerebrospinal fluid (CSF) hypocretin-1/orexin-A (Hcrt-1) immunoreactivity is a biomarker for narcolepsy type 1 (NT1). The current radioimmunoassay (RIA) does not allow precise quantification, limiting the assessment of disease severity and evolution. This study aimed to reproduce previously proposed workflows to quantify Hcrt-1 and Hcrt-2 by liquid chromatography coupled to tandem mass spectrometry (LC–MS/MS), identify RIA antibody targets, and to compare two purification methods in their ability to identify patients with NT1.

**Methods:**

Solid phase extraction (SPE) and LC–MS/MS measurements of Hcrt-1 and -2 levels from 10 NT1 patients and 21 controls were implemented based on existing literature. Immunoprecipitation (IP) with magnetic beads coupled to the RIA antibody (Phoenix) was performed and peptides identified by MS. Finally, Hcrt-1 (full-length) and Hcrt-1 fragment_1-16_ levels were analyzed in 4 patients and 4 controls prepared from both workflows by nano-LC coupled to high-resolution mass spectrometry.

**Results:**

Despite linear calibration curves for Hcrt-1 and Hcrt-2 (*R*^2^ > 0.99) could be assessed with LC–MS/MS, full-length Hcrt-1 concentrations were at the low end of the calibration curve with slightly lower levels in NT1 (*p* = 0.04). Hcrt-2 was undetectable. The discovery experiment with IP most reliably identified Hcrt-1 fragment_1-16_. SPE revealed much higher concentrations than IP. Hcrt-1_1-16_-levels allowed distinguishing patients and controls after SPE and IP, whereas full-length Hcrt-1-levels after IP did not.

**Conclusions:**

Hcrt-1_1-16_ appears stable and abundant in CSF of controls, allows identification of patients with NT1 and is a promising surrogate marker. SPE seems more suitable for further method validation in clinical routine.

**Statement of Significance**

This proof-of-concept study is a systematic approach to the mass-spectrometric quantification of the neuropeptide hypocretin-1/orexin-A (Hcrt-1) in the cerebrospinal fluid, since the currently used radioimmunoassay (RIA) does not allow precise quantification. It reveals Hcrt-1 N-terminal derived fragment_1-16_ as one main target of the RIA-antibody, and strengthens its potential role as a new surrogate marker for Hcrt-1 for targeted quantification. Hcrt-1_1-16_ is significantly reduced in patients with narcolepsy type 1 and appears to serve as a possible marker to discriminate NT1 from controls using two different purification methods, prior to measurements by nano-liquid chromatography coupled to high resolution mass spectrometry. Findings will need replication in a larger and more diverse cohort, including patients with different central disorders of hypersomnolence and intermediate RIA measured hypocretin levels to further determine their clinical significance.

## Graphical Abstract

**Graphical Abstract:**
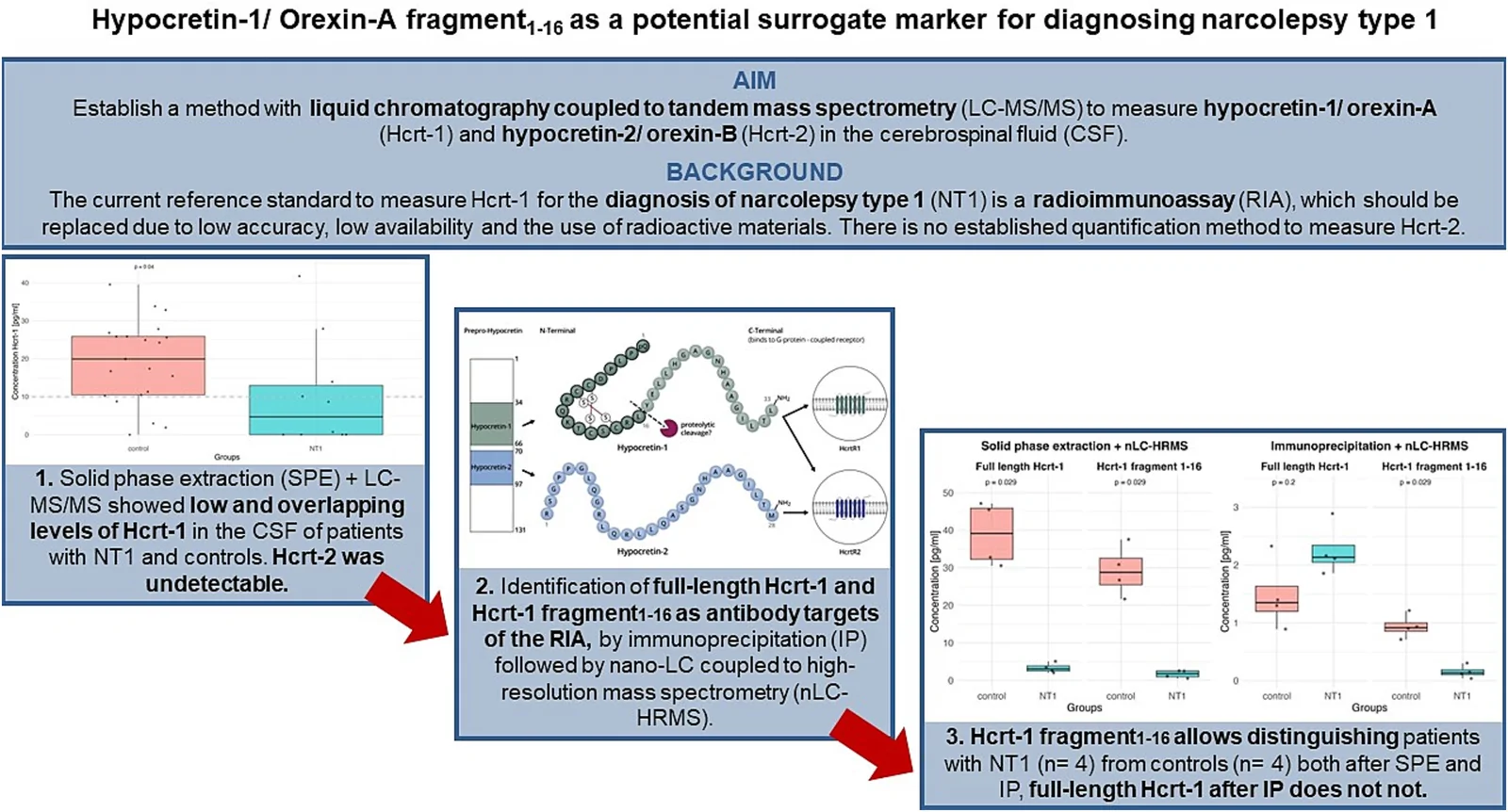

## Introduction

Following the discovery of the hypocretin/orexin system (1998) and demonstration of low cerebrospinal fluid (CSF) immunoreactivity of hypocretin-1 (Hcrt-1, also known as orexin-A) in narcolepsy (2000), the use of a radioimmunoassay (RIA) became in the last two decades the standard method for clinical use [1, 2].

This method suffers of several limitations [3]. The Hcrt-1 RIA, most commonly distributed by Phoenix Pharmaceuticals Inc. [4], uses a polyclonal antibody that can lead to cross-reactivity with binding to not only Hcrt-1, but also to unspecified fragments [5]. A recent study has systematically assessed the binding of the RIA antibody to different synthetic fragments of Hcrt-1 and hypocretin-2 (Hcrt-2, also known as orexin-B) and showed that the antibody also binds some of the Hcrt-1 fragments [6]. The antibody did not bind to Hcrt-2, corresponding to the manufacturer’s description. Batch-to-batch variability or the lack of harmonization using international standard reference material (e.g. Stanford reference material) may further impact specificity, accuracy, and inter-assay reliability [7]. The availability of the RIA is limited, and laboratories are increasingly aiming to replace it by other, non-radioactive methods [7]. There is also evidence that the redox-milieu may differ as a function of disease which could additionally affect the binding of the antibody [6].

These limitations are a challenge in clinical routine as absent or reduced Hcrt-1 levels measured by RIA (≤110 pg/mL using Stanford reference samples or <1/3 of the mean of a normal population) are most specific for the diagnosis of narcolepsy type 1 (NT1) [1, 8]. For the “narcolepsy borderland,” including narcolepsy type 2 (NT2) and idiopathic hypersomnia (IH), normal or intermediate levels of Hcrt-1 are reported [9, 10]. There is a current debate about the significance of intermediate Hcrt-1 levels and the cut-off determination using the RIA method for the diagnosis of NT1 [11]. The absence of reliable quantitative methods limits the possibility to monitor the evolution of narcolepsy and to find predictors for disease progression, as some patients with NT2 and IH might progress to NT1 during the disease course [12, 13].

The clinical significance of Hcrt-2 for sleep and other disorders is unclear, mostly because its detection in the CSF remains an obstacle [14]. It originates from the same precursor molecule, prepro-hypocretin, as Hcrt-1, and its C-terminal region has a high similarity to Hcrt-1 (Figure 1) [15]. However, compared to Hcrt-1, it has different hydrophilic properties and is missing the stabilizing disulfide bonds in the N-terminal region, which could make it more prone to degradation or stickiness with glass or plastic collecting tubes and thus complicates quantification [15].

**Figure 1:**
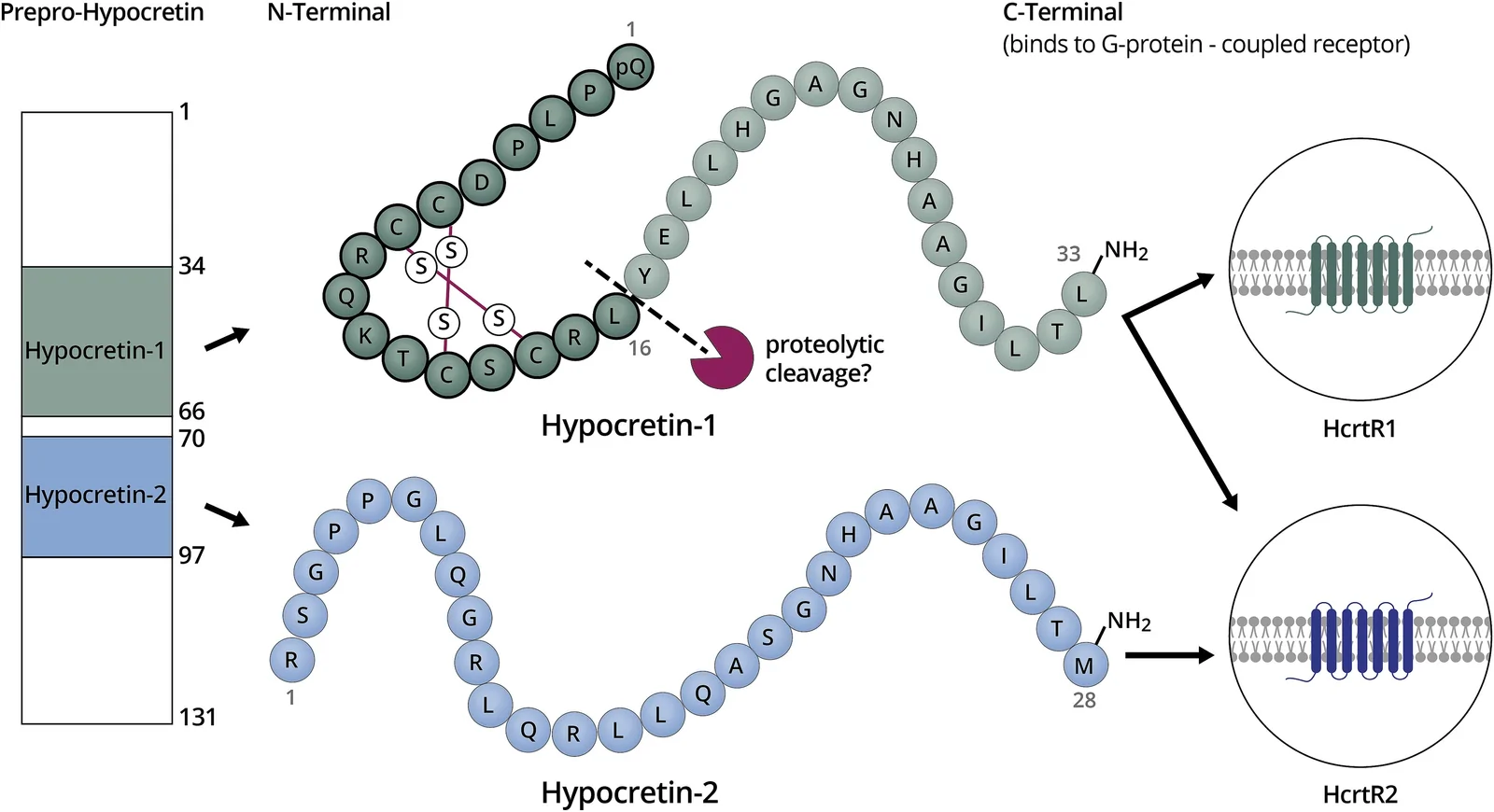
The precursor prepro-hypocretin and the resulting peptides Hcrt-1 and Hcrt-2 with their amino-acid structure. Hcrt-1 contains four cysteines, forming two stabilizing disulfide-bridges. Both Hcrt-1 and -2 show a C-terminal amidation. Consistent detection of Hcrt-1 fragment_1-16_ (darker color, corresponding to prepro-hypocretin residues 34-49) suggests proteolytic cleavage between the Leucin (L, position 49) and Tyrosin (Y, position 50), rather than simple degradation. Hcrt-1_1-16_ probably has no biological function, as it is the C-terminus of intact Hcrt-1 that binds to hypocretin-receptor 1 and hypocretin-receptor 2, both of which are G-protein coupled receptors. © *Inselspital, Bern University Hospital, Dept. of Neurology*.

The need for a reliable quantification of CSF hypocretin has given rise to the development of alternative methods, mainly to measure Hcrt-1, such as enzyme-immuno-assay and mass spectrometry (MS), none of which so far has been established for use in clinical routine. A summary of currently used tests and their limitations can be found in a recent mini-review [16].

To date, four studies have investigated MS methods for CSF Hcrt-1 [17–20] and Hcrt-2 [19]-quantification in humans. The first three reported conflicting results with generally significantly lower levels as those measured by RIA [17–19]. Specifically, Bårdsen et al. reported very low and partly overlapping levels of Hcrt-1 between patients with NT1 and controls [18]. Recently, and overlapping with the experiments for this study, a 16-mer polymer was proposed as a surrogate marker for Hcrt-1, which had previously been detected by a MS study of the CSF proteome without a specific focus on narcolepsy [20, 21]. Two other studies have used MS for the measurement of Hcrt-1 and -2 in animals, including cynomolgus monkeys and mice [22, 23]. Narita et al. described the use of a combination of anti-adsorptives to avoid adhesion to vials and prevent the loss of Hcrt-1 prior to quantification and were able to detect a molecule identified as Hcrt-2 [22]. They also described diurnal fluctuations and differences in Hcrt-1 levels depending on the sampling site (cisternal versus lumbar), which might give insights into the degradation process of Hcrt-1.

The main advantage of MS over antibody-based methods is the potential for exact quantification of peptide concentrations in body fluids. However, setup and instruments are expensive, require high operator skill and the sensitivity of the method depends on pre-analytic and analytic factors, requiring setup, and workflow optimization.

This stepwise experimental proof-of-concept study aimed to establish a workflow based on liquid chromatography coupled with MS to quantify Hcrt-1 and Hcrt-2 and to assess possible pitfalls by (1) replicating and testing previously introduced workflows [17–19], (2) identifying targets of the RIA antibody, and (3) comparing two different extraction methods to determine their ability in distinguishing patients with NT1 from controls.

## Materials and methods

### Mass spectrometry

First replication studies (part 1) with liquid chromatography coupled to tandem mass spectrometry (LC–MS/MS) were performed with a Shimadzu Nexera X2 ultra-high performance liquid chromatograph (UHPLC, Shimadzu Corporation, Kyoto, Japan) coupled to a QTRAP6500+ (Sciex, Darmstadt, Germany). The optimized mass transitions were similar to all three previous studies (Supplemental Table S1) [17–19]. Figure 2 provides a conceptual overview on LC–MS/MS. Source parameters are shown in Supplemental Table S2.

**Figure 2:**
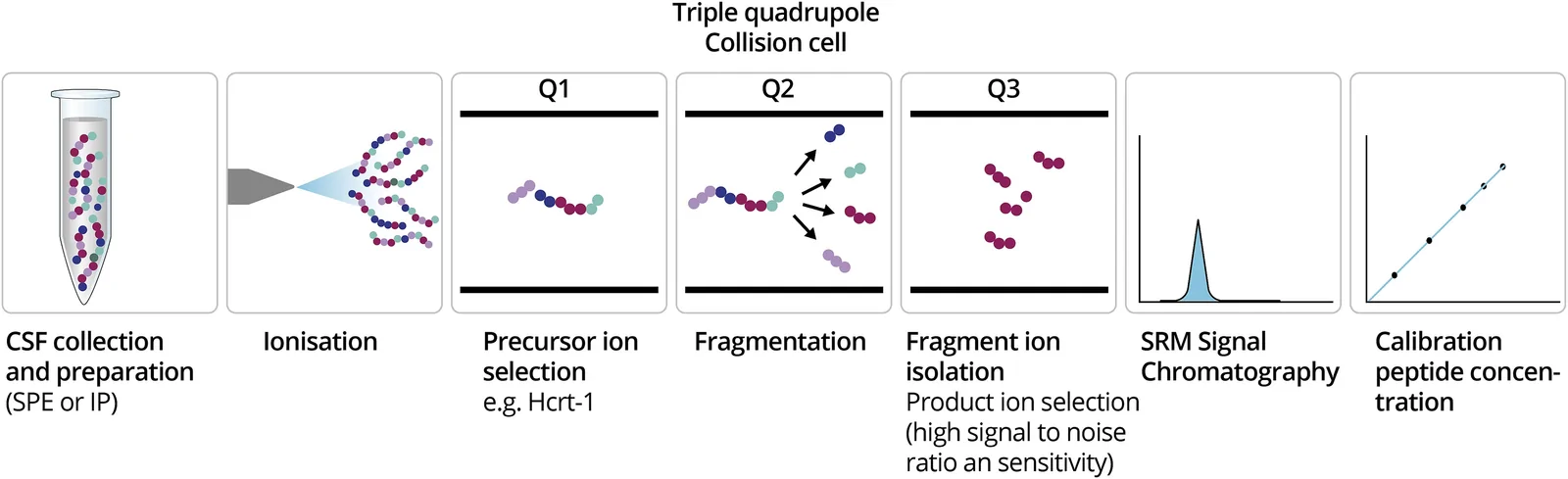
The principles of LC–MS/MS. For the replication study, we used SPE prior to LC–MS/MS, but alternatively IP could be used for purification. SMR = selective reaction monitoring. © *Inselspital, Bern University Hospital, Dept. of Neurology*.

For discovery experiments (part 2), nano-liquid chromatography coupled to high resolution mass spectrometry (nLC-HRMS) was used. At the Department of BioMedical Research (DBMR) of the University of Bern, an Orbitrap Lumos mass spectrometer (ThermoFisher Scientific, Bremen) and at the protein analysis facility (PAF) of the University of Lausanne, an Orbitrap Fusion and Exploris 480 were used for those experiments. Antibody targets were identified by a data dependent acquisition approach. Data acquisition was made in data dependent mode with precursor ion scans recorded in the Orbitrap with a resolution of 120 000 (at m/z = 250) parallel to top speed fragment spectra of the most intense precursor ions in the linear trap for a cycle time of 3 s maximum.

The MS data was searched and quantified with FragPipe using MSFragger with the Uniprot Human database (release 2024), to which common contaminants were added.

Further experiments (part 3) were performed on a Vanquish Neo UHPLC coupled with an Orbitrap Astral mass spectrometer (ThermoFisher Scientific, Bremen) at the DBMR by a targeted tandem MS approach on orexin A (numbering of amino acids based on prepro-hypocretin sequence): A.***Q[-17]***P**L[+7]**PD***C[-1]C[-1]***RQKT***C[-1]***S***C[-1]***RLYELLHGAGNHAAGI**L[+7]**T***L[-1]***.G [34, 66] (missed 3) 890.6849 (4+) and 894.1935 (4+) for the IS and Hcrt-1 fragment1-16 A.***Q[-17]***P**L[+7]**PD***C[-1]C[-1]***RQKT***C[-1]***S***C[-1]***R**L[+7]**.Y [34, 49] (missed 3) 610.6020 (3+) and 615.2801 (3+) for the IS and analyzed with Skyline 25.1.0.142 (MacCoss Lab Software, Washington).

### Solid phase extraction (parts 1 and 3)

Sample workup with solid phase extraction (SPE) was performed according to Lindström et al. [19] Synthetic standard of Hcrt-1 was purchased from Phoenix Pharmaceuticals Inc. (Burlingame, CA, USA) and its stable isotope-labeled peptide internal standard (IS) from CPC Scientific (San Jose, CA, USA). Synthetic standard of Hcrt-2 was purchased from Merck (Zug, Switzerland) and its IS from Innovagen AB (Lund, Sweden). After consistent identification of a Hcrt-1 fragment_1-16_, the synthetic peptide, containing the same two disulfide bridges like full-length Hcrt-1 (cysteine 1-cysteine 3, cysteine 2-cysteine 4), was ordered from Innovagen AB (Lund, Sweden) for part 3 of the experiments. The N-terminal harbored pyroglutamate, like full-length Hcrt-1 [full sequence: (NH2-)(Pyr)PLPDCCRQKTCSCRL(-COOH)].

Part 1: calibrations were performed for both synthetic standards of intact Hcrt-1 and Hcrt-2. Working solutions for calibration 1-9 and quality controls (QC) 1-3 were prepared in a diluting solution according to Bårdsen et al. [18] Calibration curves (10–1000 pg/mL) and QCs (50–900 pg/mL) were set up with 500 μL of pooled CSF and 50 μL of spiking solutions and 50 μL of IS of Hcrt-1 and Hcrt-2.

Briefly, 500 μL of CSF spiked with 50 μL of a mix of Hcrt-1 and -2 and 50 μL of a mix of respective IS were thoroughly vortexed. The extraction of Hcrt-1 and -2 was done using an Oasis HLB μElution Plate 30 μm (186001828BA, Waters, Milford, MA, USA). The plate was conditioned in a first step with 500 μL of methanol (MeOH), followed by a rinsing step with 500 μL of MilliQ water. The spiked CSF sample was loaded onto the plate and washed with 500 μL of water/MeOH/ammonium hydroxyde (65/30/5, v/v/v). Both hypocretins were eluted with 250 μL water/MeOH/formic acid (FA) (17/80/3, v/v/v). The samples were then dried on a Sample Concentrator with a nitrogen flow at 40°C (Techne, Cole-Palmer, IL, USA). Prior to LC–MS/MS measurements (part 1), the samples were resuspended in 50 μL water/acetonitrile (ACN)/FA (85/15/1, v/v/v), shaken for 1 min at room temperature by 1000 revolutions per minute and sonicated for 1 min. A volume of 10 μL was injected in the LC–MS system. The peptides were separated on a Shimadzu Nexera X2 (Shimadzu, Japan), mounted with a Kinetex 2.6 μm C18 100 Å (50 × 2.1 mm, Phenomenex, CA, USA) and analyzed by electrospray ionization in the positive mode with a QTRAP6500+ mass spectrometer (AB Sciex LLC, MA, USA).

For this proof-of-concept study, basic method performance characteristics were established. The limit of quantification (LOQ) approximates the limit of detection and corresponds to the lowest calibration point (Cal1 = 10 pg/mL). Based on seven independent measurement runs, the LOQ (Cal1) is estimated to have a signal to noise ratio (S/N) ≥7 and a coefficient of variation (CV) <15 per cent. According to Clinical and Laboratory Standards Institute C62-A and FDA Guidelines, acceptable criteria for the LOQ include a S/N of ≥10 and ≥5, respectively, and a maximum CV of 20 per cent [24, 25]. All results below Cal1 are only qualitative, since reproducibility, accuracy, and precision cannot be assured below this level.

To control for systematic errors, independent weighting factors were applied for calibration curves and QCs.

Part 3: for the experiments in part 3, the described SPE protocol was used, only the resuspension was adapted for the measurements by nLC-HRMS. The samples were solubilized in 25 μL H_2_O/ACN (99/1 v/v), 0.015 per cent n-dodecyl-β-D-maltosid (DDM), and 0.1 per cent FA.

Since we did not see a difference in concentration between a starting volume of 0.5 and 1 mL during the set-up phase, we chose the lower starting volume of 0.5 mL CSF for all SPE experiments. For experiments in part 3, no calibration was performed.

### Immunoprecipitation (parts 2 and 3)

Immunoprecipitation (IP) was performed with magnetic beads (MagBead, Orexin A [Human, Rat, Mouse, Porcine, Ovine, Bovine, Monkey], Phoenix Pharmaceuticals, CA, USA), coupled to the same antibody that is commonly used for the RIA (also distributed by Phoenix Pharmaceuticals Inc.). Exploratory IP experiments to discover antibody targets (part 2) were performed independently by two groups of the co-authors in two different institutes (EW/JW at the DBMR and MT at the PAF). In Bern (DBMR), first IP experiments were performed without a desalting step (Figure 3, steps 1-6), whereas in Lausanne (PAF), desalting was performed. The final protocol (part 3) was adapted from two protocols provided by Phoenix Pharmaceutical Inc., *Suggested General Method for the Extraction of Peptides from Plasma or Serum* and the IP protocol, provided together with the magnetic beads [26].

**Figure 3:**
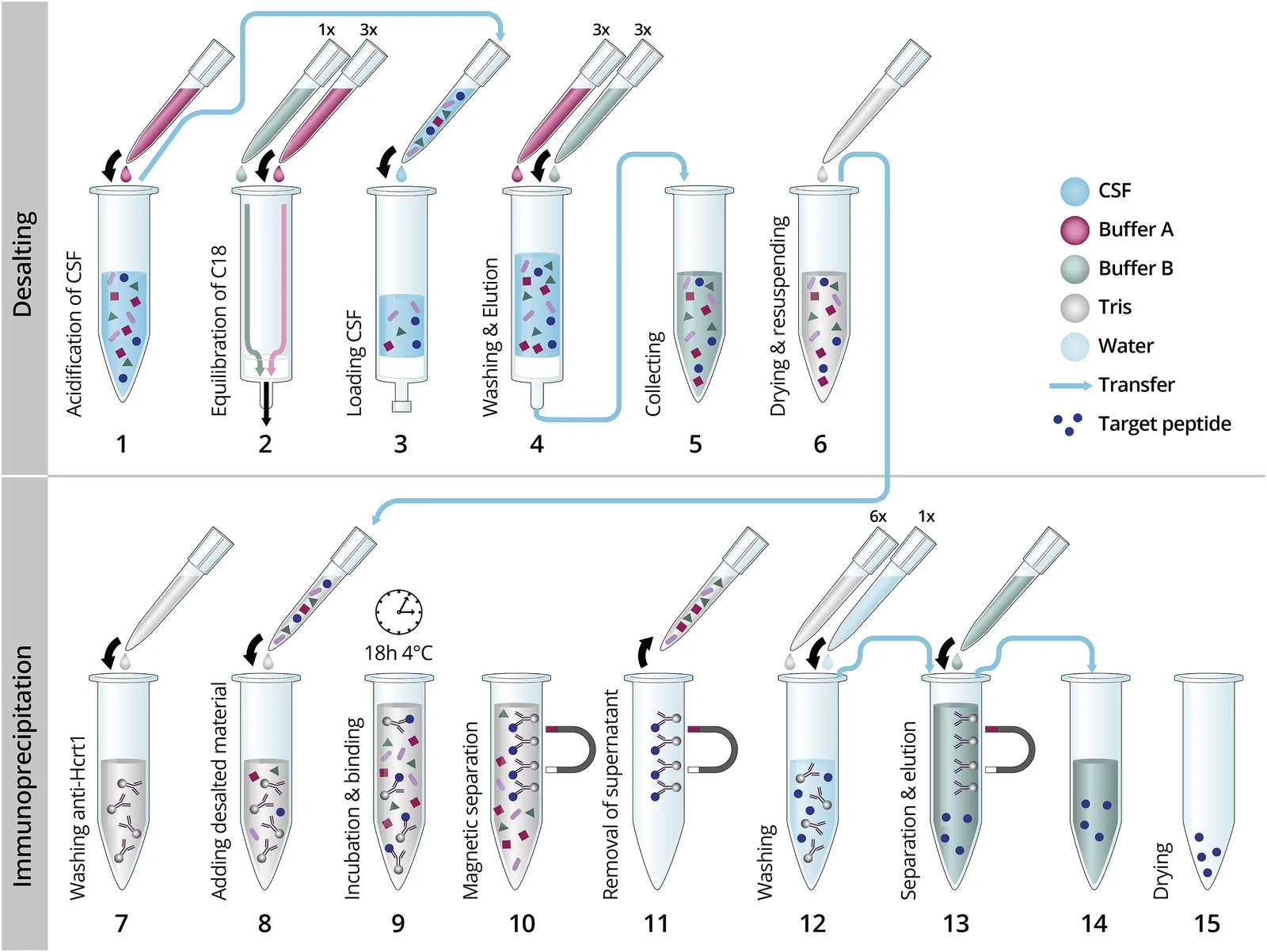
Simplified workflow of the immunoprecipitation (IP, steps 7–15) with preceding desalting procedure (steps 1–6). C18 = type of a chromatography column. (1) Acidification of 1 mL CSF with 1 mL 1 per cent trifluoroacetic acid (TFA, Buffer A). Vortex thoroughly, centrifugation over 20 min at 4°C by 17 000 rcf. (2) Equilibration of C18 SPE cartridges one time with 1 mL of 60 per cent ACN + 1 per cent TFA (Buffer B). Second equilibration step with three times 1 mL of Buffer A. (3) Loading of the full volume of desalted CSF on the SPE cartridges. (4) Three times washing with Buffer A followed by three times elution with Buffer B. (5) Collection of elution in a 5 mL reaction tube (pre-coated with 1 per cent n-dodecyl-β-D-maltosid (DDM), and washed with Buffer A). (6) Drying under nitrogen at 40°C and resuspension in 200 μL Tris buffer 250 mM pH 8. (7) Equilibration of magnetic beads for at least 30 min at room temperature. Washing of the beads with 900 μL of Tris buffer 250 mM pH 8. Fixing of reaction tubes on a magnet for 2 min and removal of supernatant. Suspension of beads in 50 μL of Tris buffer 250 mM pH 8. (8) Addition of 200 μL of desalted CSF and 660 μL of Tris buffer 250 mM pH 8 to achieve a final volume of 910 μL. (9) Incubation for 18 h at 4°C while shaken by 400 rpm. (10) Magnetic separation. (11) Removal of supernatant. (12) Washing of beads six times with 900 μL of Tris buffer 250 mM pH 8. Resolution in 200 μL MilliQ water. (13) Transfer in new 1.5 mL reaction tube (pre-coated with 0.1 per cent DDM and washed with Buffer A). Magnetic separation for 2 min, removal of supernatant. Elution by adding 60 μL of Buffer B. Incubation for 1 h at room temperature and shaking at 300 rpm. Magnetic separation for 5 min. (14) Transfer of solution with eluted peptides in reaction tube (pre-coated with 0.1 per cent DDM and washed with Buffer A). (15) Drying with nitrogen flow at 40°C. © *Inselspital, Bern University Hospital, Dept. of Neurology*.

For the IP, the samples were first desalted using Sep-Pak columns (Phenomonex Strata C18-E, 55 μM, 70 Å, 200 mg). The simplified workflow with more detailed description is presented in Figure 3 (steps 1–6). Reaction tubes were pre-coated with 1 per cent DDM. At the end of the desalting procedure, the samples were dried under nitrogen at 40°C and resuspended in 200 μLTris buffer 250 mM pH 8 (Tris).

IP was performed according to Figure 3 (steps 7–15) over 2 days, because of one incubation step lasting over 18 h.

Prior to measurements by nLC-HRMS, the samples were resolubilized in 25 μL H_2_O/ACN (99/1 v/v), 0.015 per cent DDM, 0.1 per cent FA. 2.5 μL of IS Hcrt-1 (6674 pg/mL) and Hcrt-1 fragment 1-16 (3331 pg/mL) were spiked to inject (5 μL) final concentrations of 33.4 and 16.7 pg of Hcrt-1 and Hcrt-1 fragment_1-16_, respectively.

To address recovery limitations following IP bead protocols, which frequently arise from antibody interference, low protein yield, and weak signal intensity, a starting volume of 1 mL was used (part 3). Based on evidence of binding-site competition between antibody targets and the IS in RIA tests (Supplementary material), the IS was added after the IP steps. This contrasts with SPE, when the IS was added in the beginning as usual.

### Radioimmunoassay

The RIA was performed according to the manufacturer’s description by the institute for clinical chemistry at the University Hospital in Zurich. Stanford reference samples have not been used. According to the laboratory, the calibration range is between 20 and 1280 pg/mL with a sigmoid calibration curve and a linear range between 80 and 300 pg/mL (personal communication).

### Study participants and sample collection

CSF samples used for the feasibility study (part 1), were anonymized and originated from left over material at the Center for Laboratory Medicine (University Hospital Bern, Switzerland) and a previous clinical study [27]. For exploratory experiments (not all data shown), fresh, pooled CSF from randomly sampled sources collected during clinical routine was used.

For identification of antibody targets and the final experiments (parts 2 and 3), CSF samples originated from patients with NT1 included into the international Swiss Primary Hypersomnolence and Narcolepsy Cohort Study (iSPHYNCS) at the study center in Bern, and control samples from the PCDH-study (Peptide profile in central disorders of hypersomnolence and a new method to measure hypocretin).

The iSPHYNCS (prior its internationalization in 2023 *SPHYNCS*) is an ongoing, prospective, international, multicenter cohort study currently (September 2025) including 338 individuals, with approximately 150 available CSF samples. In- and exclusion criteria were published in the study protocol [28]. CSF was collected in patients with central disorders of hypersomnolence undergoing lumbar puncture during clinical routine for exclusion of inflammation and for Hcrt-1 determination. To be included into this analysis, the availability of at least 2.5 mL of CSF, the presence of cataplexy, HLA DQB1^*^06:02 positivity and undetectable Hcrt-1 levels in the RIA (<20 pg/mL) were required. For illustrative purpose, Hcrt-1 levels of patients were set at “0.”

CSF of 35 controls was collected between 2020 and 2023 within a dedicated study protocol (PCDH study). With help of a short screening questionnaire and the Epworth sleepiness scale, we screened for excessive daytime sleepiness (EDS), hypersomnolence, sleep complaints, medication, and known sleep disorders. Patients with EDS/hypersomnolence, significant suspected or known sleep disorders, and inflammatory diseases of the central nervous system disorders were excluded. Controls were recruited during routine interventions at the department of anesthesiology and the department of neurology of the Inselspital in Bern, Switzerland. Eligible interventions for study inclusion were spinal anesthesia (mainly prior orthopedic surgeries or urological interventions) and lumbar punctures (mainly in the context of headache disorders, like migraine and idiopathic intracranial hypertension).

### Statistical analysis

Statistical analysis was performed with R (version 2023.12.1 + 402). Numerical values were represented as medians with interquartile ranges or means with ranges (for very small sample sizes). Because of small sample sizes and not normally distributed data, non-parametrical tests were performed (e.g. Wilcoxon rank sum test, for continuous data). The correlation coefficients result from a Spearman’s rank correlation. Statistical tests were performed at the 5 per cent significance level.

### Ethical approval

Anonymized CSF samples used for the feasibility study and for pooled CSF, originated from left over material at the Center for Laboratory Medicine (University Hospital Bern, Switzerland) and a previous clinical study [27]. No ethical approval was required for this trial of equipment and procedures during method development.

The iSPHYNCS and PCDH studies have obtained ethical approval (iSPHYNCS approval Switzerland: KEK 2019–00788, PCDH: KEK 2020-02615). All patients and controls from those studies undergoing lumbar puncture during clinical routine were informed and have given their written informed consent for the additional CSF-tapping for study purposes and the study participation.

## Results

### Part 1: replication of previously proposed workflows

Calibration curves for the synthetic standards of Hcrt-1 and Hcrt-2 were linear after LC–MS/MS (both *R*^2^ > 0.99), demonstrating stability of samples when spiked with synthetic controls (Supplemental Figures S1 and S2).

Four of the 10 patients with NT1 (40 per cent) and 12 out of the 21 (63 per cent) controls were female (2 controls unknown). We measured overlapping, but still significantly lower median levels of Hcrt-1 in the CSF of patients with NT1 (4.7 pg/mL [0–13.9]) compared to controls (19.9 pg/mL [10.5–25.9]) (*p* = .04) (Figure 4). The LOQ was 10 pg/mL. Lower levels (dashed line) are considered qualitative only and are shown for illustration purposes as they may still indicate the presence of Hcrt-1 but with low confidence. Because of limited residual material, it was not possible to perform the RIA for comparison. Hcrt-2 was not detectable in the CSF of patients with NT1 or controls.

**Figure 4:**
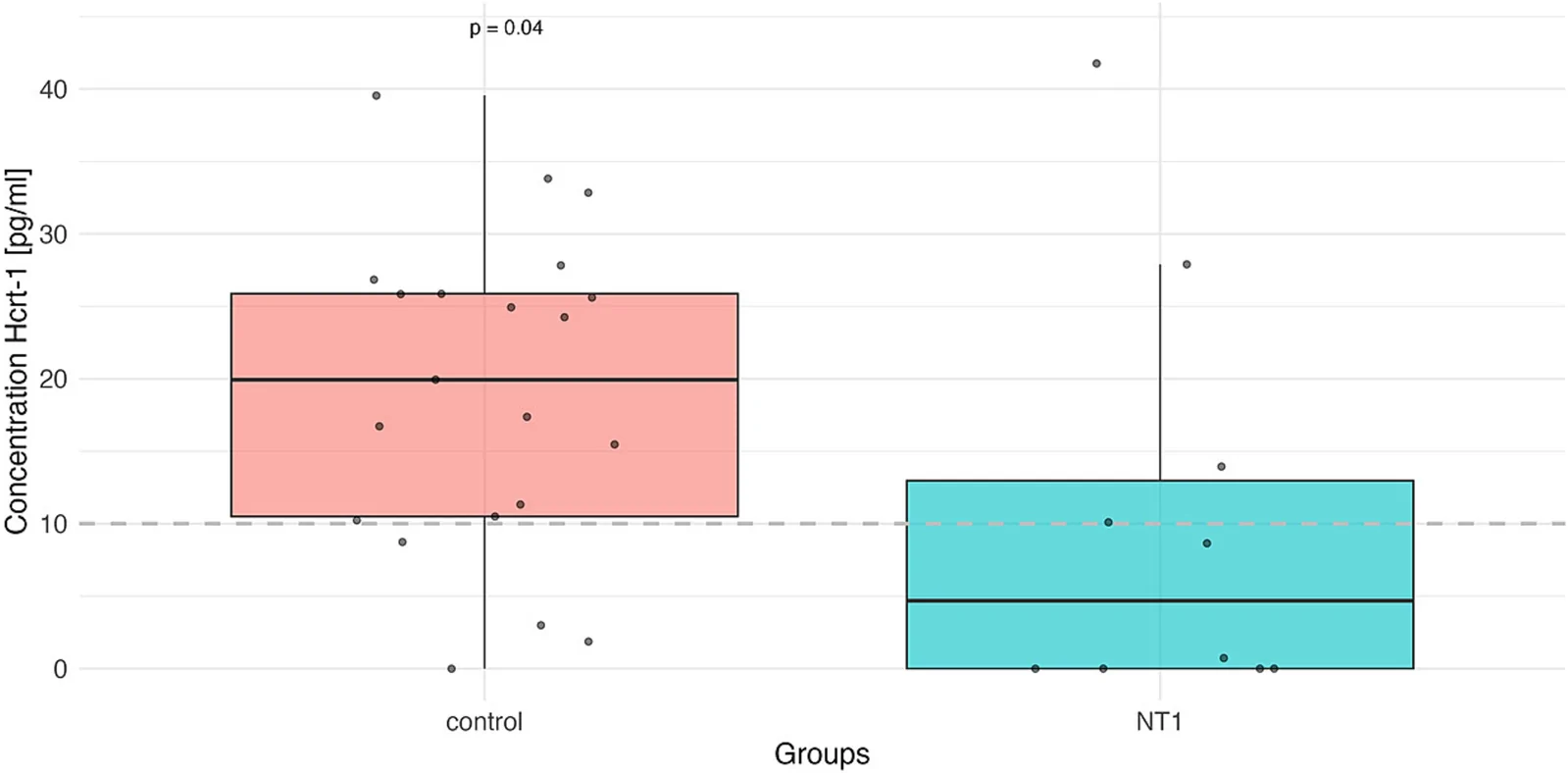
Replication of previously published workflows with SPE followed by LC–MS/MS. Hcrt-1 levels in CSF of patients with NT1 tend to be lower than in controls (*p* = .04). Levels under 10 pg/mL (dashed line) are below the LOQ. © *Inselspital, Bern University Hospital, Dept. of Neurology*.

### Part 2: identification of antibody targets of the radioimmunoassay

Initial exploratory IP experiments conducted by EW/JW (DBMR) identified a single Hcrt-1 fragment corresponding to amino acids 1-16 in a control sample with normal RIA-determined CSF Hcrt-1 levels. Separate experiments performed by MT identified full-length Hcrt-1 and several other fragments in another control patient. In contrast, CSF from a patient with NT1 showed reduced values of full-length Hcrt-1, but no other fragments. The only fragment we could reliably identify in further experiments was Hcrt-1 residues 1-16, derived from the N-terminal. Full-length Hcrt-1 was detected inconsistently and in low concentrations.

Because of reproducibility issues detecting full-length Hcrt-1 or other fragments during further IP experiments, pre-analytic steps were systematically assessed and the IP protocol provided by Phoenix Pharmaceuticals adapted accordingly (see Material and Methods and Figure 3). Various parameters were tested (e.g. glass and silanized vial and inserts, low binding plates, PBS and diluted MSG4000 as surrogate matrix, chromatographic columns, workup, etc.) without succeeding in detecting significantly higher levels of Hcrt-1. To avoid adsorption, we also tried coating with Hcrt-2 and to add citric acid and Tween 80, the latter being proposed in the literature [22]. None of these approaches has so far been successful. In addition, Tween 80 led to significant contaminations of the chromatographic columns and to signal suppression when using LC–MS instrumentation.

### Part 3: comparison of concentrations of Hcrt-1 and Hcrt-1_1-16_ between SPE and IP and between patients with NT1 and controls

Comparing SPE with IP followed by nLC-HRMS, SPE resulted in higher mean concentrations of both Hcrt-1 and Hcrt-1 fragment_1-16_ (see Table 1, Figure 5). Furthermore, after SPE, concentrations of both Hcrt-1 and Hcrt-1_1-16_ allowed clear separation of patients with NT1 and controls (both *p* = .029). After IP, concentrations of Hcrt-1_1-16_ were significantly lower in patients with NT1 compared to controls (0.15 vs. 0.94 pg/mL, *p* = .029), whereas Hcrt-1 concentrations were even higher in NT1 patients compared to controls, but this difference did not reach statistical significance (2.26 vs. 1.48 pg/mL, *p* = .2) (Figure 5). Note the generally very low concentrations of detected peptides after IP. Looking at the single samples (Figure 6), concentrations of Hcrt-1 and the Hcrt-1_1-16_ were often similar, but diverging in two controls after SPE and in all patients with NT1 after IP.

**Table 1:** Concentrations (pg/mL), peptide ratio (light versus heavy labeled peptides) and retention times of Hcrt-1 and the Hcrt-1 fragment_1-16_ in controls and patients with NT1 after SPE and IP + nLC-HRMS and signal of the RIA

**Variable**	**Control**	**NT1**	***P*-value** ^†^
	*N* = 4^*^	*N* = 4^*^	
**SPE + nLC-HRMS**			
**Hcrt-1 (pg/mL)**	**38.98 [30.55–47.13]**	**3.28 [2.01–5.05]**	**.029**
Hcrt-1 ratio light-heavy	0.58 [0.46–0.71]	0.05 [0.03–0.08]	.029
Hcrt-1 retention time (min)	23.47 [23.41–23.57]	23.54 [23.48–23.58]	.2
**Hcrt-1**_**1-16**_ **(pg/mL)**	**29.19 [21.67–37.55]**	**1.66 [0.49–2.57]**	**.029**
Hcrt-1_1-16_ ratio light-heavy	0.88 [0.65–1.13]	0.05 [0.01–0.08]	.029
Hcrt-1_1-16_ retention time (min)	13.37 [13.29–13.50]	13.48 [13.16–13.88]	>.9
**IP + nLC-HRMS**			
**Hcrt1 (pg/mL)**	**1.48 [0.89–2.33]**	**2.26 [1.86–2.89]**	**.2**
Hcrt1 ratio light-heavy	0.04 [0.03–0.07]	0.07 [0.06–0.09]	.2
Hcrt-1 retention time (min)	23.59 [23.39–23.84]	23.68 [23.57–23.80]	.9
**Hcrt-1**_**1-16**_ **(pg/mL)**	**0.94 [0.71–1.21]**	**0.15 [0.04–0.30]**	**.029**
Hcrt-1_1-16_ ratio light-heavy	0.06 [0.04–0.07]	0.02 [0.00–0.07]	.2
Hcrt-1_1-16_ retention time (min)	14.07 [13.80–14.32]	13.60 [13.32–13.77]	.029
**RIA**			
**RIA signal (pg/mL)**	**264.75 [231.00–306.00]**	**Not detectable**	**.021**

^*^Mean (min–max).

^†^Wilcoxon rank sum exact test; Wilcoxon rank sum test.

**Figure 5:**
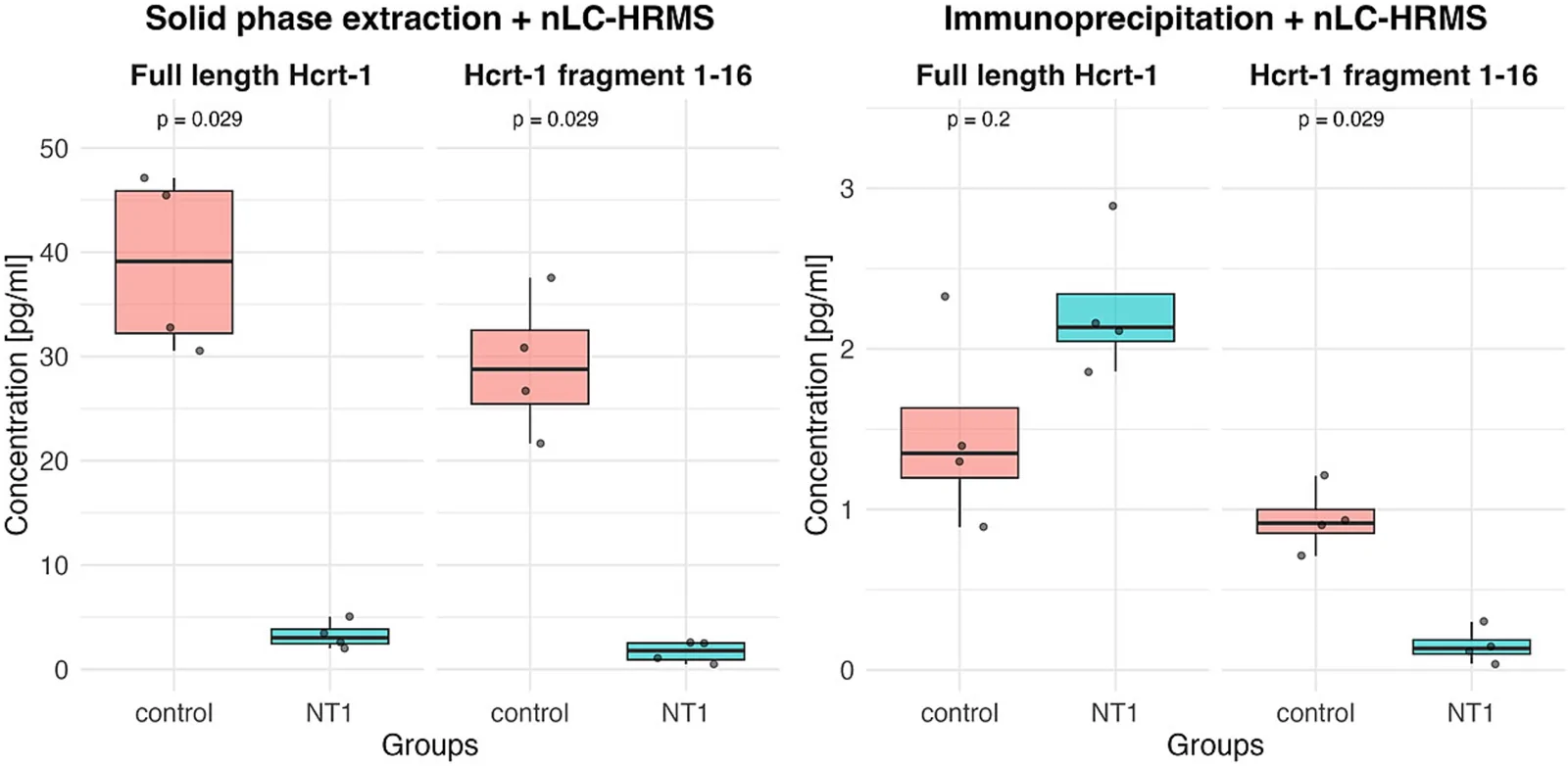
Boxplots showing the results of full-length Hcrt-1 and Hcrt-1 fragment_1-16_ measurements of four patients with NT1 and four controls, comparing SPE (left) and IP (right) prior to nLC-HRMS. Note the different concentration range of SPE and IP. © *Inselspital, Bern University Hospital, Dept. of Neurology*.

**Figure 6:**
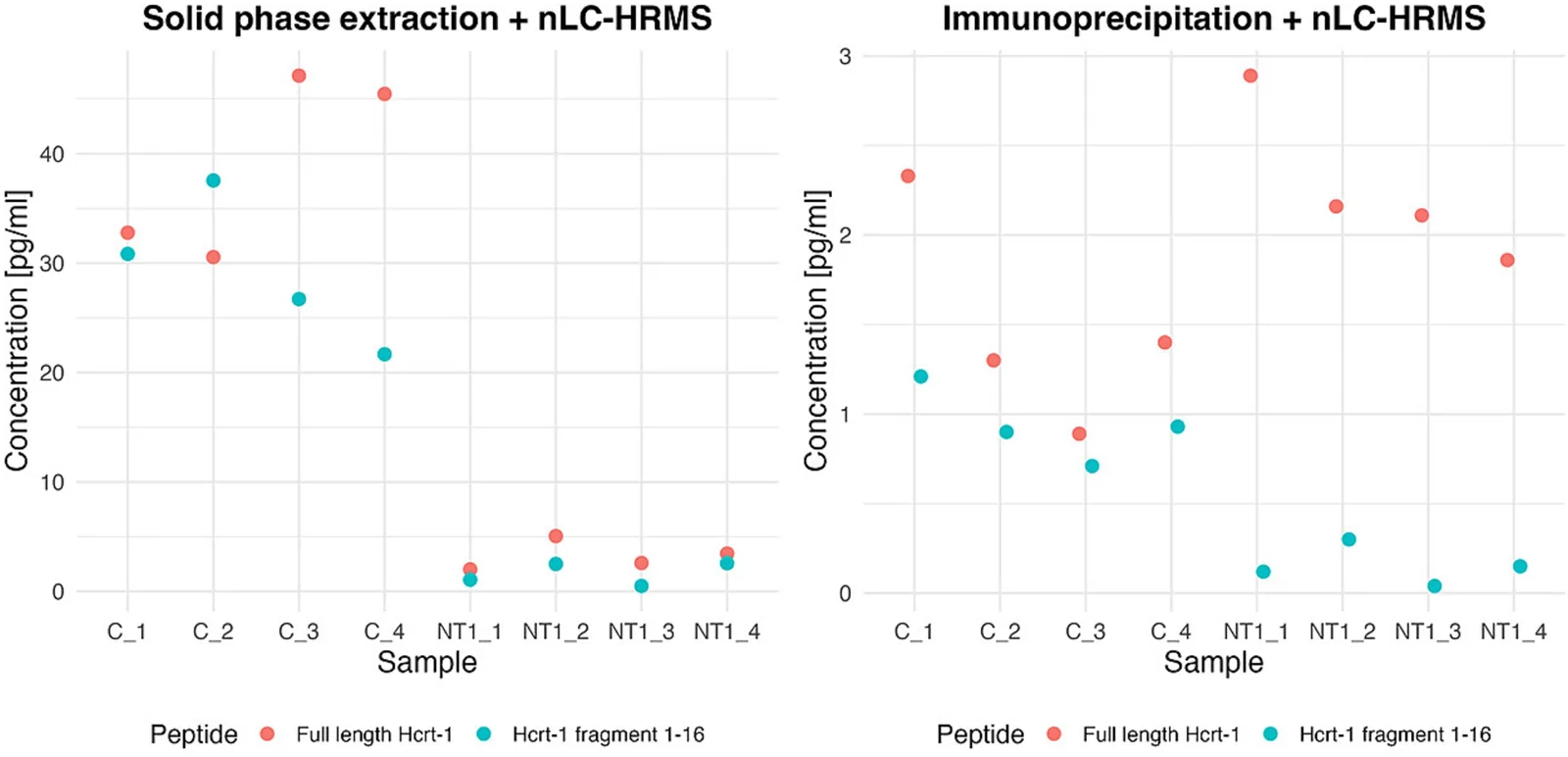
Scatterplot showing the concentrations of full-length Hcrt-1 and Hcrt-1 fragment_1-16_ per patient with NT1 (*n* = 4) and control (*n* = 4), comparing SPE (left) and IP (right) prior to nLC-HRMS. Note the different concentration range of SPE and IP. © *Inselspital, Bern University Hospital, Dept. of Neurology*.

Concentrations, retention times and the ratios between the light and the heavy labeled peptides (IS) are shown in Table 1.

Concentrations of Hcrt-1 and Hcrt-1_1-16_ after SPE + nLC-HRMS showed a significant correlation with concentrations from the RIA (*p* = .03 and *p* = .0015), demonstrating Spearman’s rank correlation coefficients of ρ = 0.76 and ρ = 0.9 respectively (Figure 7). Due to very low concentrations, we did not correlate results from IP + nLC-HRMS and RIA. RIA results are shown in Table 1.

**Figure 7:**
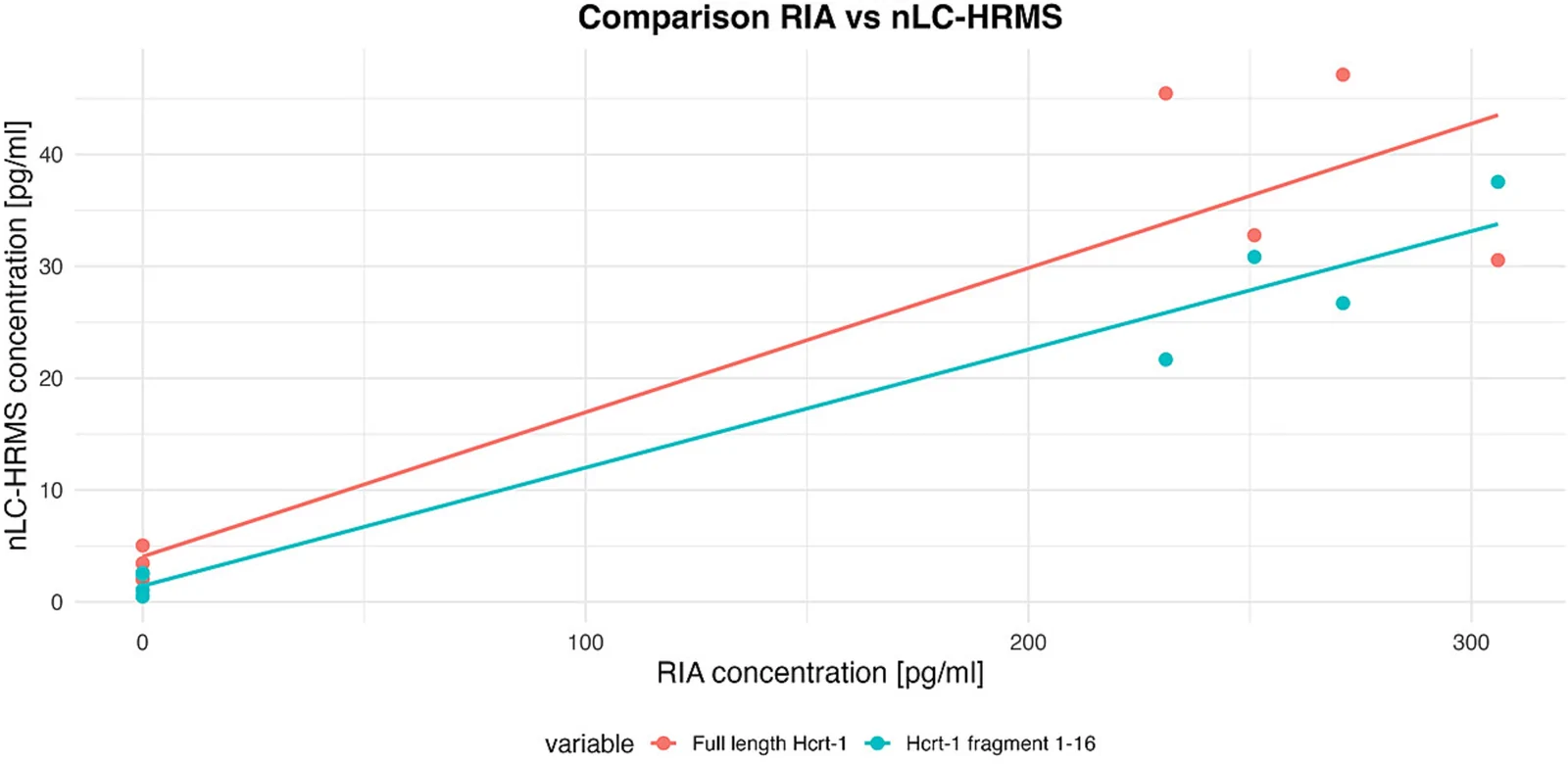
Scatterplot comparing concentrations from the RIA with SPE + nLC-HRMS. Spearman correlation between the methods is significant with Spearman’s rank correlation coefficients for Hcrt-1 of ρ = 0.76 (*p* = .03) and ρ = 0.9 (*p* = .0015) for Hcrt-1 fragment_1-16_. Note the different concentration ranges of the RIA and nLC-HRMS. © *Inselspital, Bern University Hospital, Dept. of Neurology*.

## Conclusion/Discussion

This and one previous study have identified the N-terminal derived Hcrt-1 fragment_1-16_ as a potential surrogate marker for mass spectrometric Hcrt-1 quantification by two different methods (IP with the RIA antibody; fractionation and high performance liquid chromatography [HPLC]) [20]. Both studies show evidence for its diagnostic relevance. In our study, the concentration of Hcrt-1_1-16_ allowed the differentiation of patients with NT1 from controls in a small cohort and after purification with two different methods (SPE and IP, Figures 5 and 6, Table 1). In contrast, concentrations of full-length Hcrt-1 after IP were highly variable and not significantly different between patients and controls (*p* = .2). Hcrt-1 concentrations were also low and overlapping in first replication experiments (Figure 4), and in a previous publication, which led us to investigate reasons for low sensitivity of the method [18]. However, linear calibration curves with QCs showed good recovery and no evidence for systematic error, and suggested that the developed workflow is technically performant.

Taken together, results from our replication experiments (part 1) and previous studies suggest that, in at least some patients with NT1 the concentration of full-length Hcrt-1 may be unchanged or only minimally decreased. Increasing indirect and direct evidence indicates that the reduced signal detected by RIA reflects a decrease in specific Hcrt-1 fragments rather than in full-length Hcrt-1 [5, 17–20]. This interpretation is supported by targeted mass-spectrometric studies showing partially overlapping Hcrt-1 concentrations in NT1 patients and controls [18, 19]. However, if Hcrt-1 deficiency is primarily caused by the loss of hypocretin-producing neurons in the lateral hypothalamus, a reduction in the total amount of both Hcrt1 and its fragments would be expected. If, in some patients, only the fragments were reduced, alternatively, changes in degradation processes could also lead to a reduced RIA signal.

The Hcrt-1 fragments_1-16_ and -_1-14_ (*residues 34-49 and 34-47*) were first described by Zougman et al. in 2008 [21]. In 2019, experiments by Sakai et al. suggested, that full-length Hcrt-1 might contribute only as much as 10 per cent to the RIA signal [5]. In their study, the use of a different antibody limits comparability. Very recently, Hcrt-1_1-16_ has also been detected after fractionation and separation with HPLC + microLC–MS (*orexin-A 16-mer*) [20]. In line with the work of Vialaret et al., in our study, all values for Hcrt-1_1-16_ in patients with NT1 are below their proposed cut-off of ≤10.67 pg/mL, with the limitation that we have not performed a formal calibration (part 3). The confirmation of Hcrt-1_1-16_ by different methods further increases the impact and potential value of the finding, pointing towards its stability and abundance in control persons. The replicability also suggests that this fragment might be a product of enzymatic cleavage rather than nonspecific degradation processes in the CSF (Figure 1). So far, there is little knowledge about degradation mechanisms of Hcrt-1. As both the RIA and MS are able to detect full-length Hcrt-1, however, not all Hcrt-1 might be processed to fragments [6, 17–19]. Furthermore, the detected Hcrt-1_1-16_ does not play a role in biological processes, as it is missing the C-terminal ending of Hcrt-1 that binds to the hypocretin receptors 1 and 2 (HcrtR1, HcrtR2) (Figure 1) [29].

Full-length Hcrt-1 seems not to be the ideal target for mass spectrometric quantification. Comparably low full-length Hcrt-1 levels to our first measurements were shown by Bårdsen et al. [18] Lindström et al. were able to quantify higher levels, but their measurements also showed some overlap between patients with NT1 and controls (*n* = 2 out of 44 with undetectable levels) and non-detectable levels in over half of included patients with NT2 [19]. They were also not able to detect Hcrt-2. Hirtz et al. quantified higher Hcrt-1 levels than in our study [17]. Besides the choice of the target, adsorption and loss of material during sample handling is probably relevant as well, when comparing results of the RIA to MS.

Phoenix Pharmaceuticals Inc. has published a small list of peptides to which the RIA antibody does not bind (e.g. Hcrt-1_16-33_, Hcrt-2), but it remains unclear to what epitope(s) or molecules it binds to [4]. A recent study has shown that it binds to the N-terminal region [6]. We showed in our IP experiments, that the RIA antibody binds to Hcrt-1_1-16_ and to full-length Hcrt-1. It might possibly bind to other specific and unspecific targets. During one discovery experiment using IP (part 2), few other fragments of Hcrt-1 and its precursor were identified, but could not reliably be detected during follow-up experiments. In exploratory RIA studies (Supplementary material), the addition of different concentrations of synthetic peptides to pooled CSF and buffer solutions confirmed the binding of the antibody to the synthetic standards of Hcrt-1, Hcrt-1_1-16_ and to the heavy labeled IS, but not to Hcrt-2. Furthermore, the experiments pointed towards a substantial competition between different antibody targets (Supplemental Figure S3). The affinity of the antibody might even be better for Hcrt-1_1-16_ than for full-length Hcrt-1 (Supplemental Table S3). Those findings need systematic replication but are consistent with a recent study systematically testing different synthetic fragments of Hcrt-1 by RIA, including Hcrt-1_1-16_ (reduced *orexin-A_1-16_*) [6]. Although, this study only tested reduced fragments (without intact disulfide bonds).

Qualitatively and from a practical point of view, IP is labor intensive with several time-consuming steps, including manual washing, extraction and drying, making it prone to handling errors, loss of material and more difficult to handle larger sets of samples (Figure 3). Without formal testing, we consider the method to be less reliable. Another challenge concerns the poorly defined characteristics of the polyclonal antibody. Competition among Hcrt-1 fragments with different affinities, combined with non-equilibrium binding conditions, may influence the measured concentrations in ways that is difficult to predict [6]. In an experimental set-up, we attempted to deplete CSF of all antibody-binding components by IP, followed by RIA. However, a residual RIA-signal remained, even when higher amounts of magnetic beads were added (Supplemental Table S3), indicating that IP was not fully efficient for reasons that remain unclear. Limited IP-efficiency could be another reason for low resulting concentrations after IP (Figure 5). If IP was to be further pursued for Hcrt-1 measurements, its efficiency will need to be further improved. Further reasons to explain the large difference of concentration between RIA but also between SPE and IP + nLC-HRMS (Figure 5) also need to be evaluated.

In contrast to IP, SPE is an easier procedure which can be performed within hours and at with high throughput. SPE prior to nLC-HRMS showed considerably higher concentrations of both Hcrt-1 and Hcrt-1_1-16_ compared to IP. Whether SPE + nLC-HRMS consistently reveals higher concentrations of full-length Hcrt-1 or Hcrt-1_1-16_ (Table 1) needs to be determined by including a larger sample size.

To reach higher sensitivity, we were using a nLC-HRMS system for our experiments with a 22 min gradient time. This is comparable to the acquisition time of 20 min using a microLC–MS system in a previous study [20].

The protocols provided by Phoenix Pharmaceuticals for the desalting procedure and for IP have been adapted (Figure 3) [26]. CSF, for example, is not specified in the protocol (only plasma/serum). The original protocol leaves some details unspecified, which needed to be determined during the planning phase.

After the replication study, we tested pre-analytic and analytic factors that could increase sensitivity. The use of different anti-adsorptives and materials did not increase concentrations of Hcrt-1. Moreover, Tween 80 led to significant contaminations of the chromatographic columns and to signal suppression. This makes its use obsolete for clinical practice in which a high throughput with minimum wear of material is warranted. For the IP experiments, reaction tubes were coated with DDM to minimize adsorption and tube changing steps were reduced whenever possible.

The discovery of possible competition between different antibody-targets during IP and RIA (Supplemental Figure S3) and shown by Kukkonen et al. [6], led us to add the heavy-labeled IS after the IP. Doing so, the IS allowed to have a trace where expected Hcrt-1 molecules would elute from the column (LC-MS/MS analysis of IP’s) and to quantify how much Hcrt-1 or Hcrt-1_1-16_ can be recovered with the IP, but this method does not allow correct quantification of the molecules in the CSF. However, for this proof-of-concept study, we aimed to avoid competition between the IS and the peptides in question. High concentrations of IS might have obscured the very small concentrations of Hcrt-1 and Hcrt-1_1-16_ during the experimental set-up phase.

Our first replication study (part 1) is limited using residual samples without further clinical information and without results from the RIA. The comparison between two methods, and between NT1 and controls (part 3), is limited by the small sample size but has the advantage of clinically well-defined groups. To confirm our hypothesis that Hcrt-1_1-16_ is a promising target for further MS studies, we consider the sample size as sufficient.

Results will need replication and validation (including intra- and inter-assay reliability and accuracy measures) in a larger cohort, including a more diverse spectrum of patients with central disorders of hypersomnolence and also with intermediate Hcrt-1 values by RIA.

This is the first MS study on narcolepsy and Hcrt-1 evaluating RIA antibody targets by IP and comparing two different purification methods. It is also the first study evaluating concentrations of full-length Hcrt-1 and Hcrt-1_1-16_ in a healthy control population compared to patients with NT1.

## Supplementary Material

Supplementary_material_V1_4_tp_zsag115

## Data Availability

The data that support the findings of this study are available from the corresponding author upon reasonable request.
