# Supplementary material for: Hypocretin-1/orexin-A fragment1-16 as a potential surrogate marker for diagnosing narcolepsy type 1
Source: Sleep. 2026 May 15;49(10):zsag115. doi: 10.1093/sleep/zsag115 (PMC13658270; doi:10.1093/sleep/zsag115)
Supplement: Supplementary file 1 [file supplementary_material_v1_4_tp_zsag115.docx]

# Hypocretin-1/ Orexin-A fragment_1-16_ as a potential surrogate marker for diagnosing narcolepsy type 1

Elena S. Wenz^1,2,^ Jean-Christophe Prost^3^, Sophie Braga- Lagache^4^, Gabriela M. Mäder^3^, Jan D. Warncke^1^, Martin Luginbühl^5^, Patrick Y. Wuethrich^5^, Friedrich Lersch^5^, Christoph Schankin^1^, Irina Filchenko^1^, Livia Fregolente^1,2^, Kseniia Zub^1^, the iSPHYNCS consortium, Seiji Nishino^6^, Carlo R. Largiadèr^3^, Manfred Heller^4^, Mehdi Tafti^7^, Markus H. Schmidt^1^, Claudio L. A. Bassetti^1,8^

^1^Department of Neurology, Inselspital, Bern University Hospital, University of Bern, Bern, Switzerland

^2^Graduate School of Health Sciences, University of Bern, Switzerland

^3^Department of Clinical Chemistry, Inselspital, Bern University Hospital, University of Bern, Switzerland

^4^Department for Biomedical research, Core Facility Proteomics & Mass Spectrometry, University of Bern, Switzerland

^5^Department of Anaesthesiology and Pain Medicine, Inselspital, Bern University Hospital, University of Bern, Bern, Switzerland

^6^Sleep and circadian neurobiology laboratory, School of Medicine, Stanford University, Stanford, USA

^7^Department of Biomedical Sciences, University of Lausanne, Lausanne, Switzerland

^8^Medical Faculty, University of Bern, Bern, Switzerland

Corresponding author: Elena S. Wenz, Rosenbuehlgasse 25, 3010 Bern, Switzerland, [elena.wenz@insel.ch](mailto:elena.wenz@insel.ch)

**Supplementary material**

## 1. Calibration curves (Experiments part 1)

**Figure S1:** Calibration curve for the synthetic standard of full- length Hypocretin-1 (Hcrt-1_1-33_), R^2^> 0.99.

**Figure S2:** Calibration curve for the synthetic standard of Hypocretin-2 (Hcrt-2_1-28_), R^2^> 0.99.

## 2. Mass spectrometry transitions and source parameters (Experiments part 1)

**Table S1:** Selected reaction monitoring (SRM) parameters for Hcrt-1 and Hcrt-2 and their dedicated Internal Standard (IS) (Experiments part 1).

Quant: Quantifying Transition, Qual: Qualifying Transition, CE: Collision Energy, DP: Declustering Potential, RT: Retention Time.

| **Name** | **Quant/Qual** | **Precursor (Q1) (*m/z*)** | **Fragment (Q3) (*m/z*)** | **Dwell (s)** | **CE (V)** | **DP (V)** | **RT (min)** |
| --- | --- | --- | --- | --- | --- | --- | --- |
| Hcrt-1_1-33_ | Quant | 713.06 | 854.1 | 10 | 25 | 41 | 1.18 |
| Hcrt-1_1-33_ | Qual | 891.03 | 853.9 | 10 | 37 | 141 | 1.18 |
| Hcrt-1_1-33_ IS | Quant | 894.46 | 857.4 | 10 | 38 | 101 | 1.18 |
| Hcrt-1_1-33_ IS | Qual | 894.46 | 1142.7 | 10 | 38 | 101 | 1.18 |
| Hcrt-2_1-28_ | Quant | 725.45 | 696.3 | 10 | 20 | 91 | 1.14 |
| Hcrt-2_1-28_ | Qual | 725.45 | 683.8 | 10 | 24 | 41 | 1.14 |
| Hcrt-2_1-28_ IS | Quant | 727.53 | 698.8 | 10 | 20 | 131 | 1.14 |
| Hcrt-2_1-28_ IS | Qual | 582.24 | 698.8 | 10 | 20 | 131 | 1.15 |

**Table S2:** Source Parameters (Experiments part 1).

CAD: Collisionally activated dissociation, CUR: Curtain Gas, GS1: Source Gas1, GS2: Source Gas2, IS: IonSpray Voltage, TEM: Source Temperature

| **Parameters** | **Values** |
| --- | --- |
| CAD | High |
| CUR | 35 |
| GS1 | 70 |
| GS2 | 40 |
| IS | 5500 |
| TEM | 600 |

## 3. Additional exploratory experiments

These experiments are exploratory and were performed only once, as described. They have been referred to in the discussion of the main manuscript.

### Radioimmunoassay experiments 1

**Aim**

We aimed at testing if the radioimmunoassay (RIA) antibody was detecting the synthetic standards of full-length hypocretin-1 (Hcrt-1), Hcrt-1 fragment_1-16_ and hypocretin-2 (Hcrt-2) used for the quality controls (QC), and their dedicated internal standard (IS, stable isotopically labeled peptide) and if there were differences depending on the matrix.

**Methods**

RIA was performed of solutions of pooled cerebrospinal fluid (CSF) and phosphate buffered saline (PBS), pure and spiked with different concentrations (qc1=50 pg/mL, qc2=180 pg/mL, qc3=900 pg/mL) of synthetic peptides (Hcrt-1, Hcrt-1 fragment_1-16_, Hcrt-2). Three samples with pooled CSF and three samples with PBS buffer were spiked with a mix of Hcrt-1, Hcrt-2 and Hcrt-1 fragment_1-16_ with the same increasing concentrations of each peptide (mix with qc1=50 pg/mL, qc2=180 pg/mL, qc3=900 pg/mL of each peptide). In addition, we added IS to three samples of pooled CSF. The IS contained a mix of heavy labelled Hcrt-1 and Hcrt-2 and Hcrt-1 fragment_1-16_ (500 pg/mLeach), which makes for a total concentration of 1500 pg/mL of peptide mix.

The RIA was performed according to the manufacturer’s description by the institute for clinical chemistry at the University Hospital in Zurich. Stanford reference samples have not been used. According to the laboratory, the calibration range is between 20 and 1280 pg/mL with a sigmoid calibration curve and a linear range between 80 and 300 pg/mL (personal communication). No dilutions were performed.

**Results**

As expected, samples spiked with Hcrt-2 did not produce a signal in addition to the baseline signal of the pooled CSF and were therefore used as negative controls (Supplemental Figure S3). In six pooled CSF samples (3 unspiked, 3 spiked with Hcrt-2), the RIA detected a mean baseline signal of 236 pg/mL with a low coefficient of variance (CV) of 3.7% (Supplemental Figure S3).

In the pooled CSF samples, in addition to the baseline value, increasing RIA signals were detected for increasing concentrations of the synthetic standards of Hcrt-1 and Hcrt-1_1-16_. At the lowest concentration (50pg/mL), a higher signal resulted for the fragment Hcrt-1_1-16_ compared to Hcrt-1. With higher concentrations, the signal for Hcrt-1 was higher than the signal for the fragment. A mix of Hcrt-1, -2 and the fragment Hcrt-1_1-16_ resulted in a slightly higher signal at the first two concentrations (50 pg/mL and 180 pg/mL). The result did not correspond to the sum of the concentrations of Hcrt-1 and the fragment. No RIA signal was higher than 500 pg/mL, even if added concentrations of synthetic peptides were higher than that.

The heavy labelled IS was also detected, three measurements resulting in a mean signal of 477 pg/mL (range 464 – 495 pg/mL).

 **Figure S3:** Pooled cerebrospinal fluid (CSF) samples spiked with increasing concentrations (qc1=50 pg/mL, qc2=180 pg/mL, qc3=900 pg/mL) of hypocretin-1 (Hcrt-1), hypocretin-2 (Hcrt-2), the Hcrt-1 fragment_1-16_ (fragment_1_16) and a mix of increasing concentrations of Hcrt-1, 2 and the fragment (50 pg/mL, 180 pg/mL, 900 pg/mL of each peptide respectively). The dotted line indicates the mean radioimmunoassay (RIA) signal of unspiked CSF (236 pg/mL).

### Radioimmunoassay experiments 2

**Aims**

1. To test with RIA, whether there still is an immunoreactive signal after immunoprecipitation (IP) with two different amounts of magnetic beads coupled to the RIA antibody.
2. To test whether alkylation of the synthetic standards makes a difference in the resulting RIA signal.

**Methods**

1. IP was performed of six 500 µL samples with pooled CSF- three with 50 µL and three with 100 µL of magnetic beads (Phoenix Pharmaceuticals), respectively (Supplemental table S3). After IP, for each amount of beads, one sample was left untreated, one sample was spiked with 200 pg/mL of synthetic full-length Hcrt-1 and one sample with 200 pg/mL of Hcrt-1 fragment_1-16_. Afterwards, RIA was performed.
2. Two 500 µL samples of Phosphate Buffered Saline (PBS) were spiked with 200 pg/mL of the synthetic standards of Hcrt-1 and Hcrt-1 fragment_1-16_, as provided by the manufacturer, with intact disulfide bridges. Two 500 µL PBS samples were spiked with Hcrt-1 and Hcrt-1 fragment_1-16_ after reduction and alkylation, which should lead to the breakage of the disulfide bonds. The RIA signal was determined.

**Results**

1. After IP with two different quantities of magnetic beads, the RIA still measured immunoreactivity in the unspiked samples of pooled CSF, which corresponded to 65% (50 µL beads) and 56% (100 µL beads) of the raw RIA signal of pooled CSF (236 pg/mL, see RIA experiments 1) (Supplemental Table S3). The signals of the samples spiked with the fragment Hcrt-1_1-16_ were considerably higher than the signals of the samples spiked with full-length Hcrt-1.
2. The RIA did not detect 200 pg/mL of full-length Hcrt-1 spiked in PBS (<20 pg/mL) in neither the dealkylated or the alkylated form. The signal for non-alkylated Hcrt-1_1-16_, spiked in PBS, was three times higher (137 pg/mL), compared to the alkylated form (40 pg/mL). The signal of the samples spiked with the fragment Hcrt-1_1-16_ were considerably higher than the signals of the samples spiked with full-length Hcrt-1.

**Table S3:** Radioimmunoassay (RIA) signal of pooled cerebrospinal fluid (CSF), after immunoprecipitation (IP), unspiked and spiked with 200 pg/mL of synthetic standard of Hcrt-1 and Hcrt-1 fragment_1-16_, respectively. IP was performed with different concentrations of magnetic beads (50 and 100 µL). RIA signal of Phosphate Buffered Saline (PBS), spiked with synthetic standards of Hcrt-1 and Hcrt-1 fragment_1-16_, in their provided standard form (de-alkylated, containing disulfide bridges) and after reduction and alkylation (“alkylated”).

| **No** | **Pooled CSF  (**µL**)** | **PBS (**µL**)** | **Beads (**µL**)** | **Hcrt-1  (50 ng/mL) (**µL**)** | **Hcrt-1 fragment_1-16  (50 ng/mL)  (**µL**)** | **Alkylation status** | **RIA signal (pg/mL)** |
| --- | --- | --- | --- | --- | --- | --- | --- |
| 1 | 500 |  | 50 | - | - | - | 154 |
| 2 | 500 |  | 50 | 2 |  | - | 176 |
| 3 | 500 |  | 50 | - | 2 | - | 284 |
| 4 | 500 |  | 100 | - | - | - | 133 |
| 5 | 500 |  | 100 | 2 | - | - | 154 |
| 6 | 500 |  | 100 | - | 2 | - | 273 |
| 7 |  | 500 | - | 2 | - | - | <20 |
| 8 |  | 500 | - | 2 | - | alkylated | <20 |
| 9 |  | 500 | - | - | 2 | - | 137 |
| 10 |  | 500 | - | - | 2 | alkylated | 40 |
